# Editorial: Novel *in-vitro* and *in-vivo* strategies to study pancreatic ductal adenocarcinoma progression and chemoresistance

**DOI:** 10.3389/fcell.2024.1435209

**Published:** 2024-06-10

**Authors:** Kranthi Kumar Chougoni

**Affiliations:** Keck School of Medicine and USC Norris Comprehensive Cancer Center, University of Southern California, Los Angeles, CA, United States

**Keywords:** pancreatic ductal adenocancinoma, chemoresistance, biomarker, anti-pd 1 immunotherapy, 3D organoid model

Pancreatic cancer is devastating, and a leading cause of deaths related to cancer. Despite significant efforts the 5-year survival rate post-diagnosis remains poor (∼10%). Pancreatic ductal adenocarcinoma (PDAC) is the most common type of pancreatic cancer that arises from the pancreatic ducts and accounts for more than 90% of pancreatic cancer cases. Treatment of PDAC is often thwarted by the lack of biomarkers for early detection, advancement in the disease often metastasized to the vital organs at the time of diagnosis, tumor microenvironment (TME) comprising of desmoplastic stroma, cancer-associated fibroblasts (CAF’s), immune cells and resilient tumor cells to chemotherapies (chemoresistance).

The goal of this Research Topic is to mainly highlight the studies exploring novel strategies to identify biomarkers to improve therapeutic outcomes in pancreatic cancer patients and targeting chemo resistant tumor cells. This Research Topic contains two original research articles and 2 case reports all of which provide valuable insights into understanding pancreatic cancer treatment.

Biomarkers are often utilized as valuable tools for early detection and for prediction of treatment outcomes. There is a huge unmet need to identify potential biomarkers in PDAC. A study conducted by Sari et al., investigated Laminin gene expression as a predictive biomarker for response to chemotherapies using multimodality approaches like genomics and proteomics. The results of the study show PAAD cell lines with high expression of Laminin (LM332) were resistant to chemotherapies like paclitaxel, gemcitabine but on the other hand sensitive to EGFR inhibitors like Erlotinib, afatinib, gefitinib and cetuximab. These findings provide a valuable insight into predicting patient response based on LM332 expression.

Patient derived 3D Organoids have gained immense attention as they closely mimic human disease compared to conventional 2D cell culture models grown in monolayers. PDAC is characterized by hypoxia, and 3D Organoids grown in hypoxic environment closely represent malignant traits. Study conducted by Kumano et al., validated the role of hypoxia in induction of Epithelial Mesenchymal Transition (EMT) and chemoresistance. The study’s results clearly show the influence of hypoxia on elevated Vimentin levels and decreased E-Cadherin levels as an indication of EMT, a most common phenomenon in PDAC. This was further supported by increased 5FU resistance of organoids grown in hypoxia compared to the normoxic conditions. These findings suggest the consideration of hypoxia in future research to translate the findings to human disease.

Immune check point inhibitors (ICI’s), especially anti-PD1 therapies, have shown promising efficacy in other cancers but not in PDAC except in some patients. In this case report by Li et al., demonstrated the usefulness of anti-PD-1 antibody as viable conversion therapies in two patients with unresectable advanced PDAC. These findings provide valuable insights into the challenging PDAC treatment.

Oncogene KRAS is mutated in more than 60% of pancreatic tumors, hence continuous efforts have been directed towards targeting mutant KRAS. While the success rate of monotherapies is low, combination therapies always remain attractive. Case report by Wang et al., demonstrated usefulness of combining Tyrosine Kinase Inhibitor (TKI), Anlotinib with PD-1 inhibitor and sequential GA regimen or FOLFIRINOX Chemotherapy in treatment of KRAS G12V mutated pancreatic ductal adenocarcinoma with liver metastasis. Although the study provides new hopes for treating pancreatic cancer patients, the results are limited by the sample size.

In summary, studies discussed in this editorial provide key insights into biomarker development to predict treatment outcomes, generation of hypoxic 3D organoids mimicking chemo resistant PDAC and case reports showing the usefulness of combining anti-PD-1 therapies with Tyrosine Kinase Inhibitors. Further research to completely understand the PDAC biology will help in the development of effective therapeutic strategies for the treatment of pancreatic cancer.

